# An Explorative Study on Using Carbon Nanotube-Based Superhydrophobic Self-Heating Coatings for UAV Icing Protection

**DOI:** 10.3390/molecules30173472

**Published:** 2025-08-23

**Authors:** Jincheng Wang, James Frantz, Edward Chumbley, Abdallah Samad, Hui Hu

**Affiliations:** Department of Aerospace Engineering, Iowa State University, Ames, IA 50011, USAabdallah.samad@kfupm.edu.sa (A.S.)

**Keywords:** UAV anti-/de-icing, carbon nanotube, superhydrophobic coating, heating

## Abstract

In-flight icing presents a critical safety hazard for unmanned aerial vehicles (UAVs), resulting in ice accumulation on propeller surfaces that compromise UAV aerodynamic performance and operational integrity. While hybrid anti-/de-icing systems (i.e., combining active heating with passive superhydrophobic coatings) have been developed recently to efficiently address this challenge, conventional active heating sub-systems utilized in the hybrid anti-/de-icing systems face significant limitations when applied to curved geometries of UAV propeller blades. This necessitates the development of innovative self-heating superhydrophobic coatings that can conform perfectly to complex surface topographies. Carbon-based electrothermal coatings, particularly those incorporating graphite and carbon nanotubes, represent a promising approach for ice mitigation applications. This study presents a comprehensive experimental investigation into the development and optimization of a novel self-heating carbon nanotube (CNT)-based superhydrophobic coating specifically designed for UAV icing mitigation. The coating’s anti-/de-icing efficacy was evaluated through a comprehensive experimental campaign conducted on a rotating UAV propeller under typical glaze icing conditions within an advanced icing research tunnel facility. The durability of the coating was also examined in a rain erosion test rig under the continuous high-speed impingement of water droplets. Experimental results demonstrate the successful application of the proposed sprayable self-heating superhydrophobic coating in UAV icing mitigation, providing valuable insights into the viability of CNT-based electrothermal coatings for practical UAV icing protection. This work contributes to the advancement of icing protection technologies for un-manned aerial systems operating in adverse weather conditions.

## 1. Introduction

The rapid development of unmanned aerial vehicle (UAV) technology has transformed the aviation landscape, offering unprecedented capabilities for operations in hazardous and remote environments previously inaccessible to manned aircraft. These systems provide substantial advantages across military and civilian applications through their cost-effectiveness and ability to minimize human risk exposure [1]. However, inflight icing remains a critical meteorological hazard that significantly impacts both manned aircraft [2,3,4,5,6] and UAVs [7,8,9]. This phenomenon occurs when supercooled water droplets impinge upon UAV propeller surfaces, forming ice accretions that compromise aerodynamic performance and operational safety.

UAVs exhibit heightened vulnerability to icing conditions compared to their manned counterparts due to several inherent limitations. UAVs have limited power available to overcome the additional drag caused by ice buildup, and their slower flight speeds mean they spend more time exposed to icing conditions, creating particularly challenging operational scenarios. These factors collectively render UAV operations in cold weather conditions highly problematic, with icing events documented in 25% of flights during specific military operations, substantially compromising mission effectiveness [10].

Recognizing the critical importance of cold-weather UAV operations, significant research efforts have focused on developing anti-icing and de-icing systems for UAV icing mitigation [8,11,12]. Contemporary approaches are broadly categorized into active and passive anti-/de-icing systems [13,14]. Active systems, exemplified by electrothermal heating technologies, introduce external energy to prevent ice formation [11]. While effective, these systems impose substantial energy demands on UAV power systems, typically necessitating heating only along leading edges to maintain energy efficiency. This selective heating approach, however, results in runback ice formation near trailing edges, degrading overall aerodynamic performance [15].

Conversely, passive systems leverage surface characteristics, particularly surface wettability properties, to mitigate ice formation without energy consumption. Among passive approaches, superhydrophobic coatings have demonstrated considerable promise. These surfaces exploit the "lotus leaf effect" enabling water droplets to effortlessly shed from slightly inclined surfaces exhibiting contact angles exceeding 150° [16]. By reducing friction on runback supercooled water droplets compared to hydrophilic surfaces, superhydrophobic coatings allow greater numbers of droplets to traverse propeller surfaces before freezing occurs. Nevertheless, superhydrophobic coatings alone cannot entirely prevent ice accumulation [8], particularly during extended icing exposure or at temperatures below −8 °C. Additionally, the durability limitations of superhydrophobic coatings constrain their practical implementation.

Recent advances in anti-icing materials have increasingly focused on integrating passive anti-icing strategies with active de-icing techniques to develop efficient, reliable, and energy-efficient anti-icing and de-icing systems [13]. These systems combine localized heating (e.g., electric heating elements, hot air, microwave radiation, infrared radiation, plasma, etc. [14]) near stagnation points with superhydrophobic surface treatments, enabling rapid removal of runback water droplets before refreezing occurs. However, adapting conventional hybrid systems to UAV propellers presents significant challenges due to the complex three-dimensional curved geometry of propeller surfaces, which complicates the precise installation of conventional heating films [15]. This limitation necessitates the development of self-heating superhydrophobic coatings capable of application to complex UAV propeller geometries through spray deposition.

Extensive research within the coatings community has focused on self-heating superhydrophobic coatings [17,18,19], with electrothermal coatings incorporating graphite and carbon nanotubes (CNT) showing promise due to their combined hydrophobicity and electrical conductivity. Moreover, the epoxy incorporation enhances coating durability. For example, Wu et al. [20] developed a fluorine-based superhydrophobic CNT coating with sprayable capability and high heating efficiency under low-voltage operation that is promising for UAV icing mitigation. The coating exhibits superhydrophobicity through the Cassie–Baxter mechanism by combining a rough CNT surface structure with low-surface-energy fluorine. However, their investigation focused exclusively on apparent contact angle [21] measurements, neglecting the influence of contact angle hysteresis on adhesion forces. Furthermore, their static de-icing test was limited to flat glass substrates, conditions substantially different from rotating UAV propellers experiencing significant convective heat transfer. Additionally, the chemical composition optimization required for maximum performance on a UAV propeller was not addressed.

This study addresses these critical limitations through the development and optimization of a novel self-heating carbon nanotube (CNT)-based superhydrophobic coating engineered specifically for UAV icing mitigation. The wettability and durability of the coating is carefully examined. A comprehensive anti-/de-icing campaign is conducted to evaluate the performance of the coating on a rotating propeller under glaze ice conditions. Experimental results demonstrate, for the first time, the anti-/de-icing effectiveness of a sprayable CNT-based self-heating superhydrophobic coating on a rotating UAV propeller within an icing research tunnel environment, thereby bridging the critical gap between static anti-icing tests on test plates and realistic UAV anti-/de-icing applications. The findings establish a new paradigm for UAV icing mitigation that seamlessly combines the passive anti-/de-icing system (superhydrophobic surfaces) with the active anti-/de-icing system (heating) and is applicable to arbitrary complex propeller geometry, potentially revolutionizing cold-weather UAV operations across military and civilian applications.

## 2. Results and Discussion

### 2.1. Effect of the Concentration of the Epoxy on Hydrophobicity

While superhydrophobic coatings have been extensively studied for potential ice mitigation applications, their limited mechanical durability represents a critical barrier to widespread implementation in practical environments. To address this limitation, epoxy resin was incorporated into the fluorine-modified carbon nanotube (FM-CNT) solution to enhance the mechanical durability of the resulting coating (with the fabrication details and the recipe of the solution being provided in Section 3.1). However, this approach necessitates a careful balance between durability and hydrophobic performance, as epoxy inherently lacks hydrophobic properties [22,23] and consequently diminishes the coating’s superhydrophobic characteristics upon incorporation.

**Figure 1:**
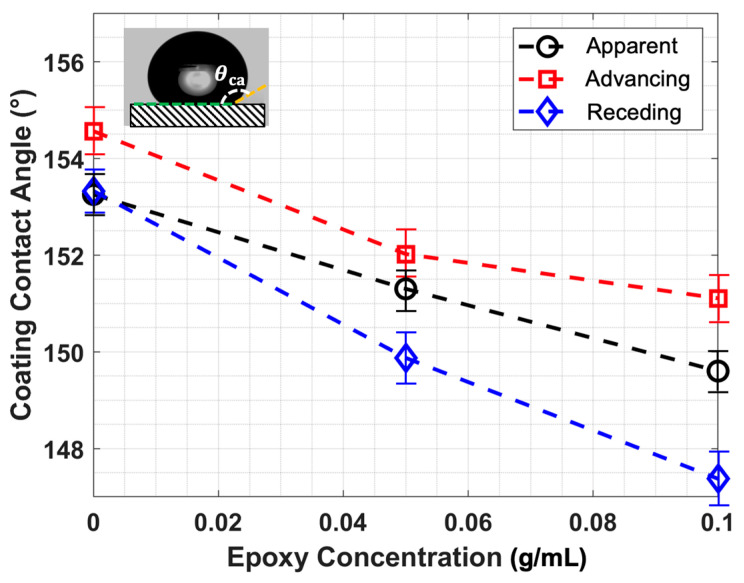
The wettability measurements for the FM-CNT coating with different epoxy concentrations.

To optimize this trade-off, the influence of epoxy concentration (i.e., the ratio of epoxy mass to FM-CNT solution volume) on coating wettability was systematically investigated through comprehensive contact angle measurements, including apparent, advancing, and receding contact angles (methodology detailed in Section 3.2). As illustrated in Figure 1, both apparent contact angle and contact angle hysteresis (i.e., the difference between the advancing contact angle and receding contact angle) exhibit nearly linear relationships with epoxy concentration. Statistical analysis was conducted using five replicate specimens to establish measurement uncertainty. At an epoxy concentration of 0.1 g/mL, the coating maintains an apparent contact angle approaching 150° with hysteresis of approximately 4°, representing an optimal compromise between superhydrophobic performance and mechanical durability. This concentration was subsequently selected for the anti-/de-icing experiment in the icing research tunnel.

### 2.2. Effect of the Concentration of the CNT on Electrical Resistance of FM-CNT Coating

The inherent non-conductive nature of epoxy results in an increase in electrical resistance with increasing epoxy concentration, presenting challenges for UAV applications where low-voltage battery systems necessitate minimal electrical resistance pathways. To address this limitation, increasing the CNT concentration emerges as a viable mitigation strategy. The electrical resistivity (ρ=ξ×A/L) of coatings deposited on glass substrates (fabrication details provided in Section 3.1) was systematically evaluated across three distinct CNT-to-epoxy mass ratios, where ξ represents the electrical resistance, A denotes the cross-sectional area of the coating, and L corresponds to the coating length.

Experimental results revealed a pronounced nonlinear relationship between CNT concentration and electrical resistivity. Specifically, mass ratios of 0.08, 0.125, and 0.25 yielded resistivities of 125 kΩ·cm, 935 Ω·cm, and 49 Ω·cm, respectively, demonstrating the critical influence of CNT concentration on the conductive electrical network formation in the coating. Given that small-scale UAVs typically operate with battery output voltages, achieving sufficiently low electrical resistance is paramount for effective power delivery in anti-/de-icing applications. Consequently, the optimal CNT-to-epoxy mass ratio of 0.25 was selected for coating fabrication in subsequent anti-/de-icing experiments conducted on rotating propellers within the icing research tunnel.

### 2.3. Heating Efficiency of the FM-CNT Coating

**Figure 2:**
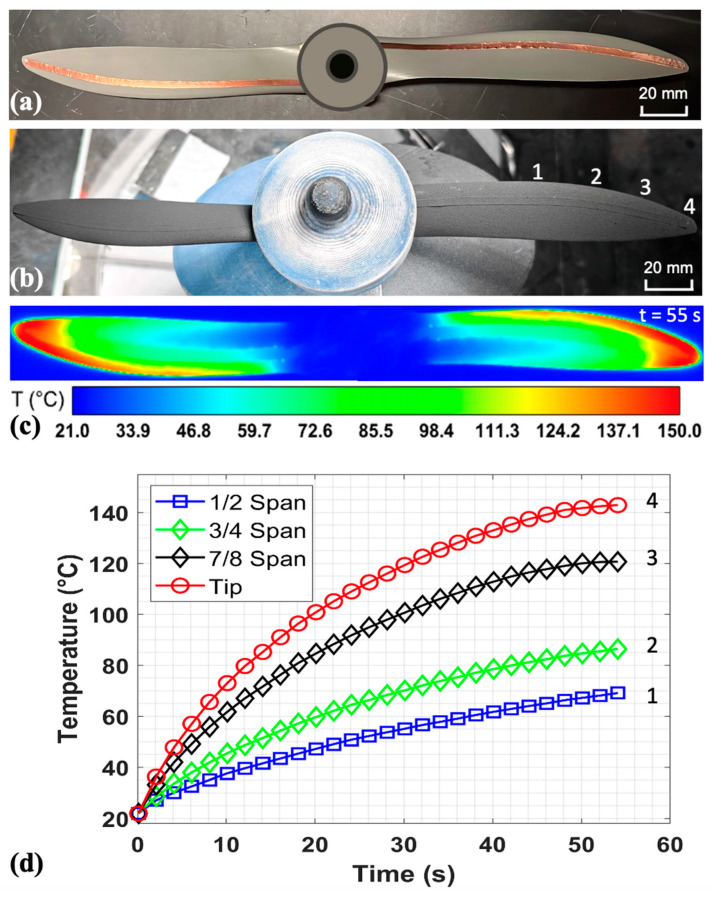
(**a**) The optimized energy-saving copper wire arrangement, which lies close to the leading edge and the tip. (**b**) The CNT-coated propeller that is installed on the advanced slip-ring system within the test section of the icing research tunnel. (**c**) The temperature distribution of the propeller coated by the CNT-coating with an electrical voltage of 24 Volts at t = 55 s. (**d**) The transient temperature measurement of selected points near the leading edge of the propeller after heating. Locations 1, 2, 3, and 4 are indicated in part (**b**), corresponding to the points at mid span, 3/4 span, 7/8 span, and tip of the blade, respectively.

To assess the thermal performance and heating efficiency of the conductive FM-CNT coating system, experiments were conducted on a full-scale UAV propeller under room-temperature conditions using an infrared camera. The test specimen consisted of an APC 11 × 7 Sport UAV propeller featuring Clark-Y airfoil geometry. As shown in Figure 2a, the electrode configuration comprised two electrically isolated copper tape strips (3 mm width) positioned on the pressure and suction surfaces of each blade, subsequently interconnected through the conductive FM-CNT coating. More details of the layout of the electrodes on the propeller and the experimental setup can be found in Section 3.1. The electrode placement was strategically optimized to concentrate thermal energy near the leading edge and blade tip regions, where ice accretion typically occurs most severely in icing conditions. Specifically, the copper electrodes were positioned proximally to the leading edge with inter-electrode spacing decreasing spanwise from one-fifth chord length at the root to 1 mm at the tip, thereby creating a non-uniform resistance distribution to achieve preferential heating in critical regions.

Figure 2b illustrates the propeller surface morphology following application of the FM-CNT coating solution. The propeller was installed in the test section of the icing research tunnel, with the hub featuring a specialized design to provide waterproof protection for the electronics located near the propeller root. The propeller was then heated using a 24 V DC power supply, yielding a power consumption of 13.7 W with a total resistance of 42 Ω. Figure 2c shows the thermal imaging after 55 s of heating confirmed the intended non-uniform temperature distribution, with higher temperatures observed at the leading edge and tip regions than the trailing edge. This thermal gradient results from the variable inter-electrode spacing, which creates higher current density and corresponding Joule heating in regions with reduced electrode separation. The observed heating pattern aligns with UAV icing mitigation requirements, as it prioritizes thermal energy delivery to areas most susceptible to ice formation while minimizing overall power consumption compared to uniform heating strategies.

The temporal temperature profiles at selected leading-edge measurement points are presented in Figure 2d, demonstrating characteristic rapid initial thermal transients followed by asymptotic convergence to steady-state equilibrium temperatures. Additionally, a pronounced spanwise temperature gradient was observed, with tip temperatures exceeding root temperatures due to the progressive reduction in inter-electrode spacing from root to tip. This behavior demonstrates the effectiveness of the variable-spacing electrode design in achieving targeted thermal management for UAV anti-/de-icing applications.

### 2.4. Anti-/De-Icing Test for a Rotating UAV Propeller Coated with FM-CNT Coating in the Icing Research Tunnel

To assess the viability of FM-CNT coating as an anti-/de-icing solution for UAV applications, comprehensive experimental investigations were performed using an FM-CNT-coated UAV propeller in Iowa State University’s icing research tunnel (ISU-IRT).Continuous electrical power transmission to the FM-CNT coating during propeller rotation was achieved through an advanced slip-ring system (detailed in Section 3.4), enabling real-time thermal anti-/de-icing capabilities. Propeller rotational speed was monitored using a tachometer and synchronized with both the delay generator and high-speed imaging system to achieve phase-locked visualization (detailed in Section 3.5), ensuring consistent propeller positioning during high-speed camera acquisition.

Experimental conditions were established to simulate representative glaze icing scenarios encountered by UAV propellers in atmospheric flights. The test environment maintained a static temperature of −5 °C, liquid water content (LWC) of 1.0 g/m^3^, freestream velocity of 10 m/s, and propeller rotational speed of 3500 rpm. These parameters were selected to simulate realistic UAV operational conditions under glaze icing conditions, providing a controlled environment for systematic evaluation of the FM-CNT coating’s anti-/de-icing performance. More details about the experimental setup can be found in Section 3.3.

**Figure 3:**
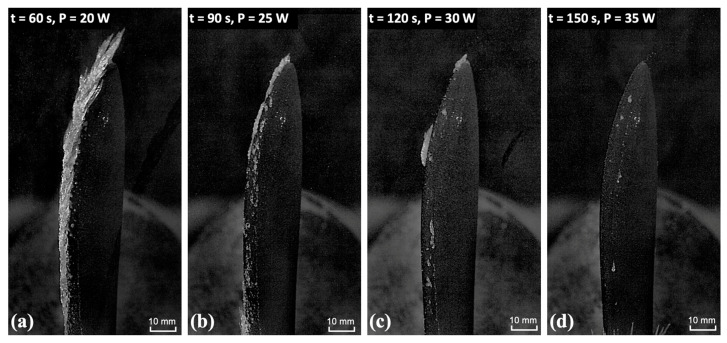
Rising-power heating test for the rotating propeller coated with the FM-CNT coating in ISU-IRT under glaze icing conditions. Four different heating powers have been tested, i.e., (**a**) P = 20 W, (**b**) P = 25 W, (**c**) P = 30 W, (**d**) P = 35 W. Time zero corresponds to the exact moment when the spray system in the icing tunnel is turned on.

A systematic rising-power heating test was conducted to determine the minimum heating power required for the coated UAV propeller to achieve ice-free operation under glaze icing conditions in the IST-IRT facility. The experimental protocol consisted of an initial 60-second preheating phase at P = 15 W, followed by activation of a supercooled water droplet spray within the ISU-IRT chamber. Subsequently, heating power was incrementally increased at 30-second intervals across four discrete power levels: 20 W, 25 W, 30 W, and 35 W. The results, presented in Figure 3, demonstrate distinct ice accretion regimes as a function of applied power. As shown in Figure 3a, the heat flux generated by the FM-CNT coating proved insufficient to achieve ice removal from the rotating propeller surface when P = 20 W. Within the intermediate power range of 25 W < P < 30 W (i.e., Figure 3b,c), partial melting of accreted ice was observed, indicating transitional de-icing behavior. In Figure 3d, complete ice prevention was achieved at P ≥ 35 W, wherein thermal energy input exceeded the latent heat energy required for ice formation. These findings establish that heating power exceeding 35 W constitutes the minimum threshold for maintaining ice-free conditions on the rotating propeller under the prescribed glaze icing environment.

To validate the findings from the rising-power heating experiments and establish a comprehensive comparison of ice accretion behavior on the rotating propeller under heated and unheated conditions, comparative experiments were conducted in the ISU-IRT facility under identical glaze icing conditions. Based on the optimization results from the rising-power heating tests, a heating power of 38 W (corresponding to 40 V) was selected for the heated configuration. The experimental protocol consisted of a 60-second preheating phase followed by a simultaneous activation of the supercooled water droplet spray and high-speed imaging systems, with anti-icing testing conducted for an additional 128 seconds under continuous heating. The unheated experiment maintained identical conditions with heating being disabled.

**Figure 4:**
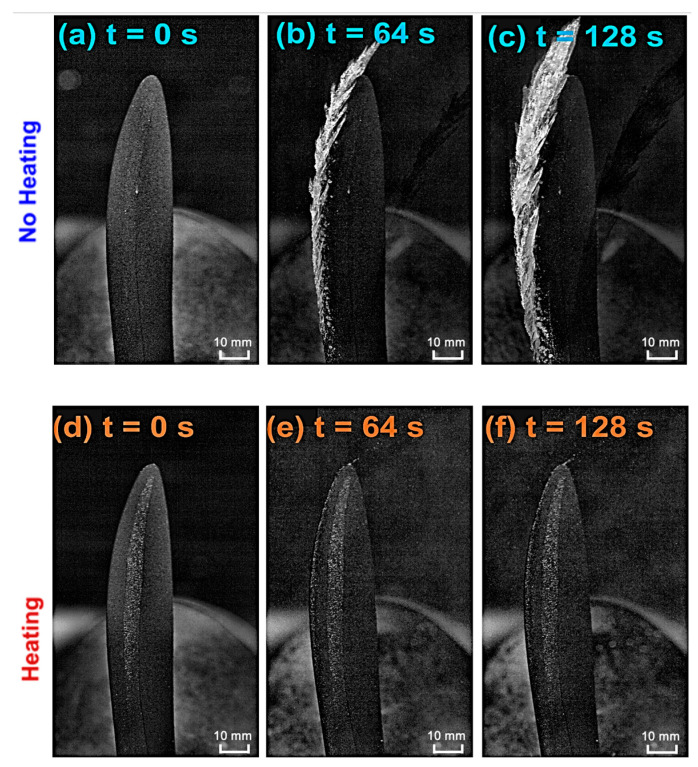
Ice accretion on a rotating FM-CNT-coated UAV propeller under glaze icing conditions. Comparison of ice formation without heating (**a**–**c**) and with heating (**d**–**f**) at sequential time intervals: t = 0 s (**a**,**d**), t = 64 s (**b**,**e**), and t = 128 s (**c**,**f**). Time t = 0 s corresponds to the initiation of the spray system in the icing research tunnel.

The effectiveness of the FM-CNT coating for UAV anti-icing applications is demonstrated in Figure 4, which reveals that the heated coating successfully maintains ice-free conditions on the UAV propeller throughout the glaze icing exposure (Figure 4a–c), while progressive ice accumulation occurs on the unheated rotating propeller (Figure 4d–f). Notably, Figure 4e,f illustrate that supercooled water droplets are effectively heated and transported by the FM-CNT coating, preventing ice formation and facilitating droplet shedding from the propeller tip through centrifugal forces.

**Figure 5:**
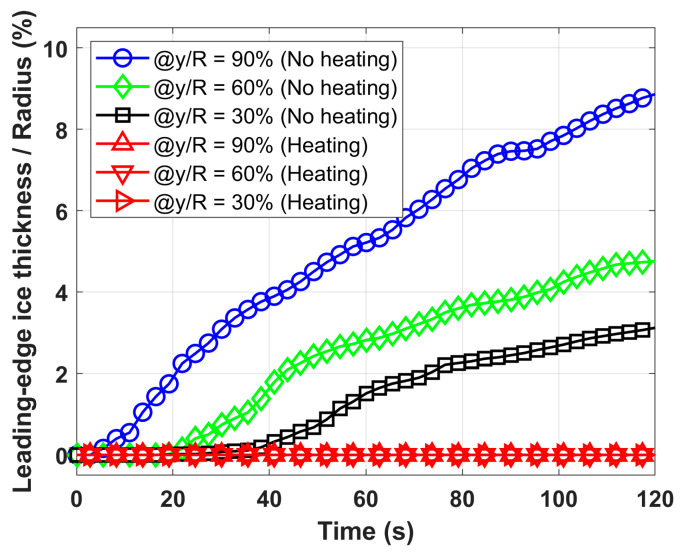
Comparison of leading-edge ice thickness between unheated and heated propellers (P = 38 W) at various blade span positions. Ice thickness is measured as the perpendicular distance from the blade’s leading edge to the ice layer’s outer boundary (i.e., the leftmost edge of the ice in Figure 4) at a given span level, normalized by propeller radius (R). The spanwise position (y) represents the distance from the propeller hub to each measurement point along the blade. The symbol "@" denotes "at," indicating the specific spanwise location where measurements were taken.

Quantitative analysis of the dynamic ice accretion process is presented in Figure 5, which depicts the temporal evolution of leading-edge ice thickness at three spanwise locations (30%, 60%, and 90% span) for both heated and unheated configurations. Leading-edge ice thickness is defined as the ice accumulation normal to the propeller’s leading-edge surface (i.e., the distance between the leading edge and the leftmost edge of ice in Figure 4 at a given span level), normalized by the propeller radius. The results demonstrate that the heated propeller maintains zero ice thickness across the entire span, while the unheated propeller exhibits progressive ice growth over time. A significant finding is the spanwise variation in ice thickness, with greater accumulation observed from root to tip, attributed to the increasing collection efficiency of supercooled water droplets from root to tip. These experimental results provide compelling evidence for the effectiveness of FM-CNT coatings in preventing ice formation on UAV propellers under glaze icing conditions.

### 2.5. Durability Test of the FM-CNT Coating by High-Speed Droplet Impingement

A durability assessment represents a critical parameter in determining the viability of the coatings for real-world applications. Given that conventional durability testing protocols employed in previous studies (including tape peeling, sandpaper abrasion, and wheel wear tests [20]) present insufficient severity to adequately simulate the operational conditions encountered by high-speed rotating UAV propellers, an accelerated high-speed droplet impingement test apparatus was implemented to rigorously evaluate the FN-CNT coating performance under intensified erosive conditions (detailed methodology provided in Section 3.6). The test apparatus consisted of a pipe-shaped wind tunnel wherein water droplets were atomized through a precision nozzle and subsequently accelerated via high-speed airflow generated by a centrifugal fan motor, resulting in controlled impingement upon square test specimens coated with the FM-CNT formulation. Operational parameters for the durability test were established at 70 m/s wind velocity and 16 g/m^3^ liquid water content (LWC), which substantially exceed the erosive intensity of typical UAV operational environments and standard icing wind tunnel protocols. This accelerated testing methodology was specifically designed to induce rapid coating degradation, thereby enabling comprehensive durability characterization within compressed timeframes.

**Figure 6:**
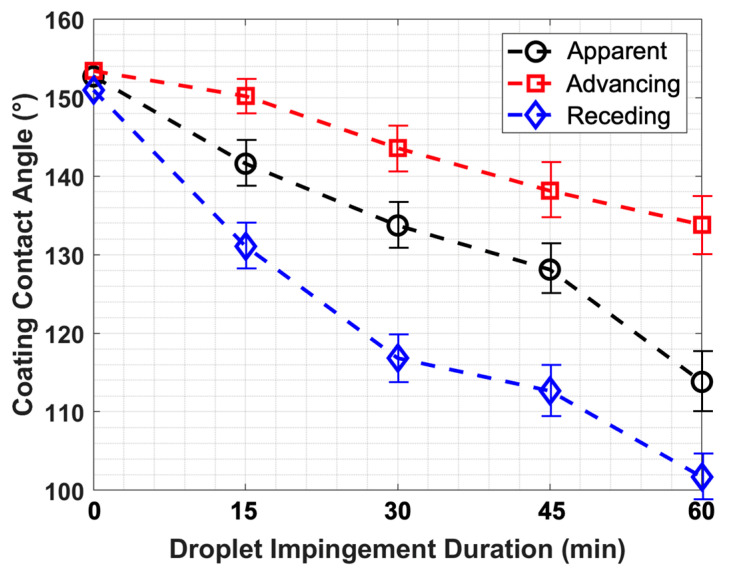
Durability test for the FM-CNT coating by high-speed droplet impingement.

The FM-CNT coating was subjected to 60 min of continuous high-speed droplet impingement testing, with wettability degradation monitored as a function of exposure duration (Figure 6). Statistical analysis was conducted using five replicate specimens to establish measurement uncertainty. Results demonstrate that while the FM-CNT coating exhibited progressive hydrophobic deterioration under continuous droplet impingement, hydrophobic characteristics (i.e., contact angle > 100°) were retained throughout the entire 60 min test period. Since the FM-CNT coating exhibits superhydrophobicity through the Cassie–Baxter mechanism, where microscale surface structures create air pockets that provide water-repelling properties, the observed degradation in the water contact angle following droplet impingement results from the morphological changes to these surface structures caused by the mechanical impact of the droplets. These findings indicate exceptional durability performance of the optimized FM-CNT coating, suggesting that the coating would demonstrate significantly extended service life under the comparatively milder erosive conditions characteristic of actual UAV operational environments in adverse weather scenarios.

## 3. Materials and Methods

### 3.1. Fabrication of the FM-CNT Coating

**Figure 7:**
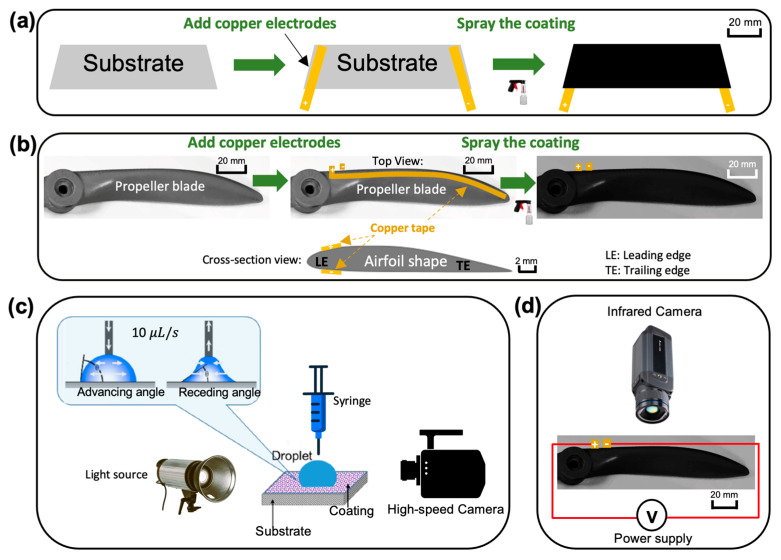
Schematic of the (**a**) fabrication process of the FM-CNT coating on a test plate, (**b**) fabrication process of the FM-CNT coating on a propeller, (**c**) wettability measuring system for the FM-CNT coating, and (**d**) infrared thermal imaging system for the heated FM-CNT coating on the propeller.

The fabrication procedure for the fluorine-modified carbon nanotube (FM-CNT) coating is illustrated in Figure 7a,b. The FM-CNT suspension was synthesized following an optimized protocol detailed in Section 2.1 and Section 2.2. Multi-walled carbon nanotubes (10 g, >95% purity, 10–20 nm diameter, purchased from US Research Nanomaterials, Inc., Houston, TX, USA) were dispersed in a solution containing 13 mL ammonia water, 26 mL deionized water, and 400 mL ethanol via ultrasonication (Q500 Sonicator, purchased from Qsonica L.L.C., Newtown, CT, USA) for 15 min. Subsequently, 1H,1H,2H,2H-perfluorooctyltrichlorosilane (PFDTES, 10 g, purchased from Thermo Fisher Scientific Inc., Allentown, PA, USA) was added to the dispersion. The mixture was stirred continuously for 24 h to facilitate the reaction between PFDTES and CNTs, resulting in a fluorine-modified CNT suspension. Prior to coating application, epoxy resin (5 g, purchased from Loctite, Rocky Hill, CT, USA) and curing agent (5 g, purchased from Loctite, Rocky Hill, CT, USA) were incorporated into 50 mL of the FM-CNT suspension, yielding an epoxy mass concentration of 0.10 g/mL as determined in Section 2.1. The final coating solution was homogenized by stirring for 15 min before application.

Substrate preparation varies according to the intended application. For wettability test specimens (glass sheets, 25 mm × 40 mm × 1 mm), copper tape electrodes were positioned at opposite ends to enable electrical connection to the power supply (Figure 7a). For UAV propeller blade testing, copper tape electrodes were installed on both the pressure and suction surfaces near the leading edge (Figure 7b). The coating solution was applied to substrates/propellers using spray depositions to achieve uniform coverage. Coated specimens/propellers were subsequently cured in a preheated oven at 60 °C for 24 h. A target coating thickness of approximately 200 μm was maintained across all specimens to ensure adequate surface coverage while preserving the aerodynamic characteristics of the propeller geometry.

### 3.2. Measurement Systems for the Coating’s Wettability and Heating Characterization

The wettability properties of the FM-CNT coating were evaluated using a custom-built contact angle measurement system (Figure 7c). Controlled-volume droplets were dispensed onto coated test substrates using a digital syringe pump (GennieTouch^TM^, purchased from Kent Scientific, Inc., Torrington, CT, USA). Droplet profiles were captured using a high-speed imaging system comprising a digital camera (FASTCAM MINI UX100, purchased from Photron, Inc., San Diego, CA, USA) and LED illumination (RPS Studio CooLED, purchased from Dot Line Corp, Humbracht Circle Bartlett, IL, USA). This configuration enabled the measurement of apparent, advancing, and receding contact angles. For apparent contact angle measurements, droplet volume was maintained at 10 μL. Advancing and receding contact angle measurements were performed at controlled infusion and withdrawal rates of 10 μL/s. Contact angles were determined from high-speed images using in-house image processing algorithms developed in MATLAB R2024a.

The thermal performance of the heated FM-CNT-coated propeller was characterized by using infrared thermography (Figure 7d). Surface temperature distributions were monitored using an infrared camera (FLIR A655SC, purchased from Teledyne FLIR LLC, Wilsonville, OR, USA) operating at a 10 Hz frame rate. Temperature measurements were calibrated against thermocouple readings obtained from the same heated region to ensure measurement accuracy.

### 3.3. Icing Research Tunnel for an Anti-/De-Icing Test of the FM-CNT Coating

**Figure 8:**
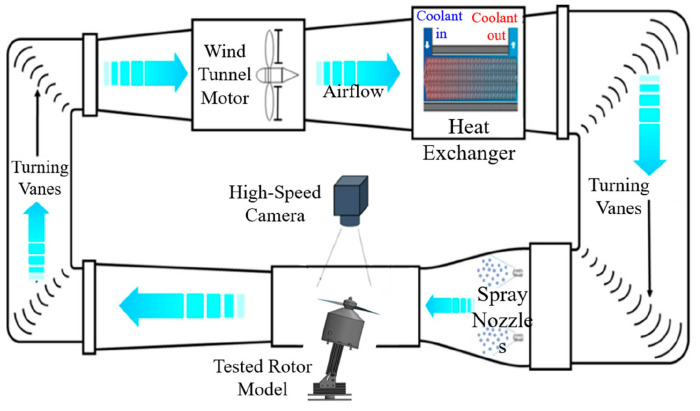
A schematic of the Iowa State University’s Icing Research Tunnel (ISU-IRT) equipped with the propeller model.

Anti-/de-icing experiments were conducted using Iowa State University’s Icing Research Tunnel (ISU-IRT), as shown in Figure 8. The ISU-IRT is a recently renovated multifunctional facility capable of replicating atmospheric icing phenomena across a wide range of environmental conditions. The tunnel features a test section with dimensions of 0.4 m × 0.4 m × 2.0 m and operates with wind speeds ranging from 5 to 100 m/s and temperatures from −25 °C to +20 °C. Supercooled water droplets with diameters between 10 and 100 μm can be generated, with liquid water content (LWC) controllable from 0.1 to 5.0 g/m^3^.

The test section was equipped with an advanced high-speed slip-ring rotor propeller system (see Section 3.4) to provide electrical power for propeller rotation and heating of the FM-CNT coating during anti-/de-icing tests. Ice accretion and anti-/de-icing performance were recorded using a high-speed camera positioned above the test section. To simulate forward flight conditions of unmanned aerial vehicles (UAVs), the propeller rotation plane was inclined at 15° relative to the horizontal. This experimental configuration has been validated in previous investigations [24].

### 3.4. Slip-Ring Rotor Propeller System

**Figure 9:**
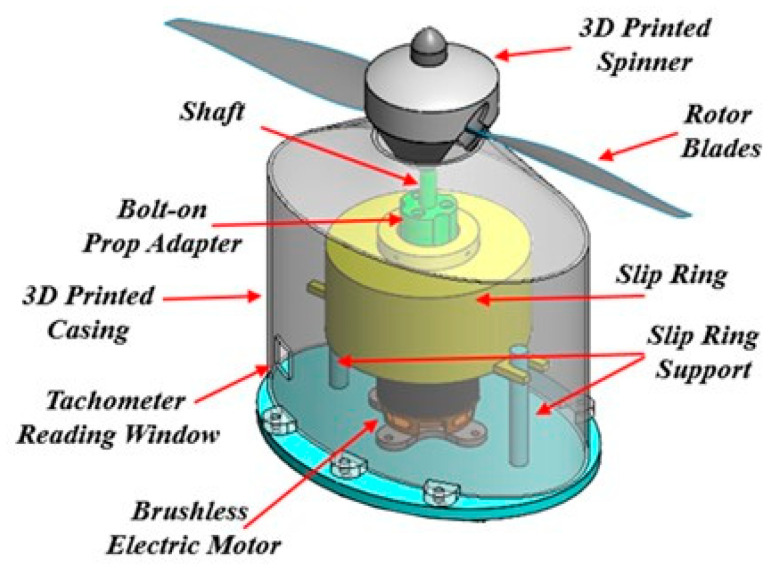
The advanced high-speed slip-ring rotor system for powering the FM-CNT coating and the propeller.

To enable continuous power delivery to the FM-CNT coating during propeller rotation, a specialized slip-ring system was implemented to prevent wire entanglement and maintain electrical connectivity. A custom high-speed slip-ring rotor blade system was developed at Iowa State University specifically for heating the FM-CNT coating on the rotating propeller assembly (Figure 9). The system comprises three primary components: a rotor propeller, a brushless motor, and an electrical slip ring (model LPT030-040, purchased from Jinpat Electronics, NE Seattle, WA, USA). This configuration ensures an uninterrupted electrical connection while preventing cable damage during operation.

The slip-ring system was engineered to operate at rotational speeds up to 4000 rpm and features a modular design for straightforward integration into icing research wind tunnels. Additionally, the system incorporates synchronization capabilities with high-speed imaging equipment, enabling phase-locked image acquisition of the rotating propeller for detailed quantitative analysis. The experimental propeller utilized was a standard APC 11 × 7 Sport UAV propeller featuring a Clark-Y airfoil profile with an 11-inch diameter, selected for its widespread use in comparable research applications.

### 3.5. Phase-Lock Imaging System for the Rotating Propeller

**Figure 10:**
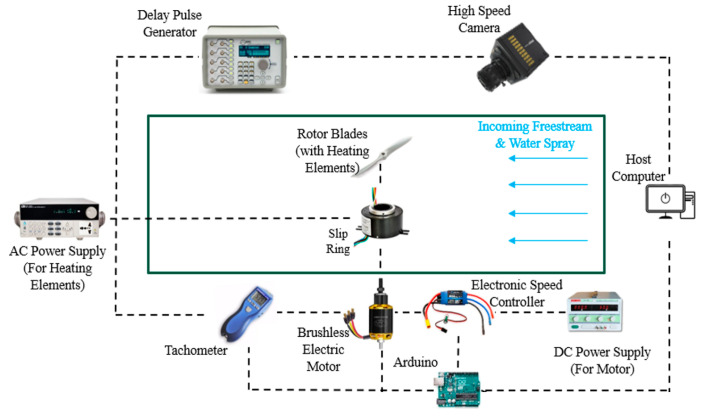
Phase-lock imaging system for the rotating propeller.

To visualize ice accretion on the rotating propeller at fixed phase angles, a phase-locked imaging system was developed (Figure 10). The system comprised three primary components: a tachometer (Omega HHT13, purchased from DwyerOmega, Michigan City, IN, USA), a delay generator (Model 575, purchased from Berkeley Nucleonics Corp, San Rafael, CA, USA), and a high-speed camera. The tachometer continuously monitored propeller rotation and generated a trigger pulse once per revolution. This pulse was transmitted to the delay generator, which produced a synchronized trigger signal for the high-speed camera. The propeller phase angle captured in each image could be precisely adjusted by modifying the delay time in the generator.

The tachometer signal simultaneously provided feedback to a closed-loop proportional–integral–derivative (PID) controller that regulated the propeller rotational speed. The PID control algorithm was implemented on an Arduino microcontroller (Uno Rev3, purchased from Arduino LLC, Boston, MA, USA), which processed real-time speed measurements from the tachometer and transmitted control signals to an electronic speed controller. The propeller rotational speed was maintained at 3,500 rpm for this study.

### 3.6. High-Speed Droplet Impingement Wind Tunnel for Durability Test

**Figure 11:**
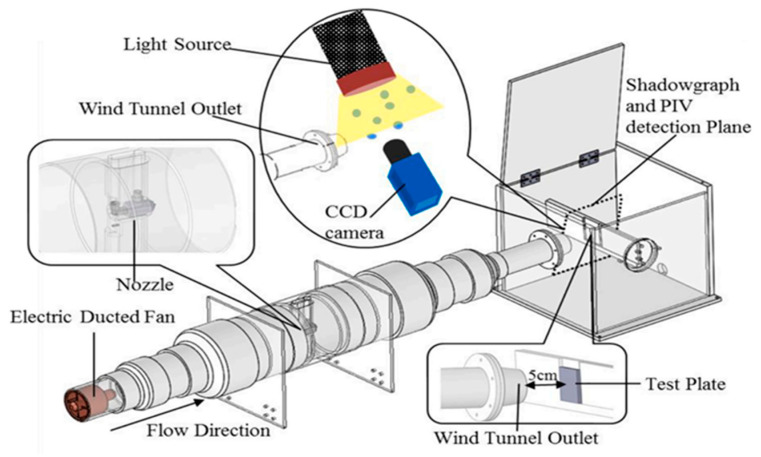
The schematic of the high-speed droplet impingement test rig for durability test of the FM-CNT coating.

Supercooled water droplet impingement represents the primary mechanism driving UAV propeller icing phenomena, posing significant challenges to coating durability. Therefore, the assessment of FM-CNT coating performance under high-speed droplet impingement conditions is essential for UAV icing application validation. A custom high-speed droplet impingement wind tunnel was constructed to simulate these operational conditions (Figure 11).

The experimental apparatus comprised a high-thrust electric ducted fan motor (EDF, purchased from JP Hobby Europe, Bourbonne les Bains, France) positioned at the tunnel inlet to generate the primary airflow. A precision nozzle assembly was installed downstream of the motor to produce water droplets with a median volume diameter (MVD) of 20 μm. Test specimens coated with FM-CNT were mounted vertically near the tunnel outlet within the droplet impingement zone.

Droplet velocities at the tunnel outlet were quantified using particle image velocimetry (PIV) [25], achieving maximum velocities of 100 m/s at full motor power. Liquid water content (LWC) was controlled through nozzle flow rate adjustment, with a maximum achievable LWC of 20 g/m^3^. This experimental configuration has been previously validated for the durability assessment of superhydrophobic and icephobic coating systems [26].

## 4. Conclusions

This study presents a novel fluorine-modified carbon nanotube-based self-heating superhydrophobic coating specifically engineered for UAV icing mitigation applications. Through a comprehensive characterization using advanced wettability measurement systems and thermal imaging, the coating demonstrated exceptional superhydrophobic properties and heating efficiency. Sprayable self-heating coatings enable conformal applications to complex geometries, including highly curved propeller surfaces, thereby addressing a critical limitation of conventional heating films. To accommodate the low-voltage power requirements of small-scale UAVs, the coating’s electrical resistance was systematically optimized through the precise control of the carbon nanotube concentration. Strategic placement of copper wire conductors ensures maximum temperature generation at critical ice-prone regions, specifically the tip and leading edge of coated propellers, resulting in enhanced energy efficiency. The coating’s anti-/de-icing performance was validated through comprehensive testing on rotating UAV propellers under glaze icing conditions in an icing research tunnel. Durability assessment was conducted through rigorous droplet impingement experiments specifically designed to simulate realistic operational scenarios encountered by UAV propellers in adverse weather conditions. The results demonstrate the coating’s robust performance under intensive droplet impingement, confirming its suitability for practical applications. These findings establish the first successful application of sprayable superhydrophobic self-heating coatings for UAV icing mitigation in an icing research tunnel, representing a significant advancement in ice protection technologies for UAVs. This work provides a foundation for enhanced UAV operational capabilities in adverse weather conditions and opens new avenues for research in shape-adaptive anti-/de-icing systems for unmanned aerial platforms.

## Data Availability

The dataset used in this study can be made available upon request from the authors.
